# Effect of a novel mesh design and the sandblasting technique on the bond strength of computer-designed and three-dimension laser printed resin bonded bridges: an in vitro study

**DOI:** 10.1038/s41598-024-59199-w

**Published:** 2024-04-10

**Authors:** Mariam Diab, Mawia Karkoutly, Shaza kanout, Jihad Abou Nassar

**Affiliations:** 1 Department of Fixed Prosthodontics, Faculty of Dentistry, Damascus University, Damascus, Syrian Arab Republic; 2 Department of Pediatric Dentistry, Faculty of Dentistry, Damascus University, Damascus, Syrian Arab Republic

**Keywords:** Health care, Medical research

## Abstract

Resin-bonded bridges (RBBs) are a minimally invasive and aesthetically pleasing treatment modality. However, their frequent failure has posed challenges for both dental professionals and patients. This necessitates the exploration of innovative strategies to enhance the longevity of RBBs. This study aimed to assess the bond strength of a mesh bridge fabricated using computer-aided design and three-dimensional (3D) printing technology in comparison to the traditional aluminum oxide sandblasting method. A total of 48 lower incisors were embedded in acrylic bases according to a standardized computer-generated model to receive 24 metal RBBs. The two groups underwent distinct metal surface treatments: the 3D mesh novel design and sandblasting with aluminum oxide particles sized at 250.00 µm. The bond strength of the bridges was evaluated, and statistical analysis was performed using the independent samples t-test with a significance level set at α = 0.05. The findings revealed a significant difference between the two methods (p < 0.001). The 3D mesh design exhibited a mean bond strength of 387.89 ± 24.15 N, while the sandblasting technique yielded a mean value of 161.46 ± 31.25 N. In summary, the 3D mesh design substantially enhanced the bond strength of RBBs compared to the traditional sandblasting technique.

## Introduction

With the evolution of dental methodologies and materials, there has been a surge in interest in identifying optimal approaches for replacing missing teeth while minimizing the removal of tooth structure, particularly in scenarios where neighboring teeth are free of decay^1^. Resin-bonded bridges (RBBs) have emerged as a standard treatment modality in the field of restorative dentistry^2^ and offer a feasible alternative to conventional bridgework and partial dentures^3^. Various enhancements have been implemented in the design and materials of resin-bonded bridges to improve their retention capabilities. The Rochette bridge, introduced in 1970, featured perforations in its metal framework that extended to the outer surface. These perforations were created using a tungsten carbide bur on the metal structure or by drilling holes in the wax before casting^4^. However, these perforations exposed the cement to saliva and compromised the strength of the metal framework, resulting in diminished retention^5^.

Progress in the field of restorative dentistry has led to advancements in RBBs through techniques like electrochemical etching, known as the Maryland Bridge, chemical etching, the lost salt technique, meshwork bridge, and sandblasting. The primary goal of utilizing RBBs is to replace missing teeth in a minimally invasive manner. These prostheses are particularly beneficial when dental implants are not suitable or traditional bridges are deemed overly invasive, especially in cases where neighboring teeth are healthy. Additionally, RBBs offer advantages such as easy application, reversibility, no need for anesthesia, cost-effectiveness, and minimal risk of pulp irritation^6^.

However, RBBs have shown a notable incidence of failure^3^. Therefore, different bridge configurations have been devised to enhance retention, prompting the need for comparative analysis against a conventional method to determine the optimal strategy for bolstering retention and ensuring sustained efficacy. Consequently, this study sought to evaluate the bond strength of a computer-aided design mesh bridge fabricated utilizing three-dimensional (3D) laser printing technology in comparison to the aluminum oxide sandblasting technique.

## Materials and methods

### Study design

This comparative in vitro experimental study was conducted at the Department of Fixed Prosthodontics, Faculty of Dentistry, Damascus University, from September 2023 to November 2023. Ethical approval was obtained from the Local Ethics Committee of Damascus University (N2780), and the study adhered to CRIS Guidelines (Checklist for Reporting In-Vitro Studies)^7^. Written informed consent was obtained from patients for tooth donation, and human lower incisors were extracted due to periodontal diseases and orthodontic reasons. The sample size was determined based on the following parameters: effect size of 0.42 (effect size f = 0.42), two-tailed 5% significance level (α = 0.05), 95% confidence interval, 80% statistical power (1-β err prob = 0.80), and 2 experimental groups. A sample size of 48 specimens was obtained, and the effect size was calculated according to a pilot study. Sound incisors free from caries, cracks, fractures, restorations, and prostheses were selected for the study, while teeth with dental fluorosis, malformations, and prior endodontic treatment were excluded. The selected incisors were similar in size^8^. The selected incisors were cleaned and immersed in a 0.5% chloramine T solution at room temperature for a week before experimentation^9^.

### Randomization and blinding

Simple randomization method was applied using the randomization online software https://www.randomizer.org/. The outcome assessor was blinded to groups allocation.

### Procedures

#### Teeth casting

**Figure 1:**
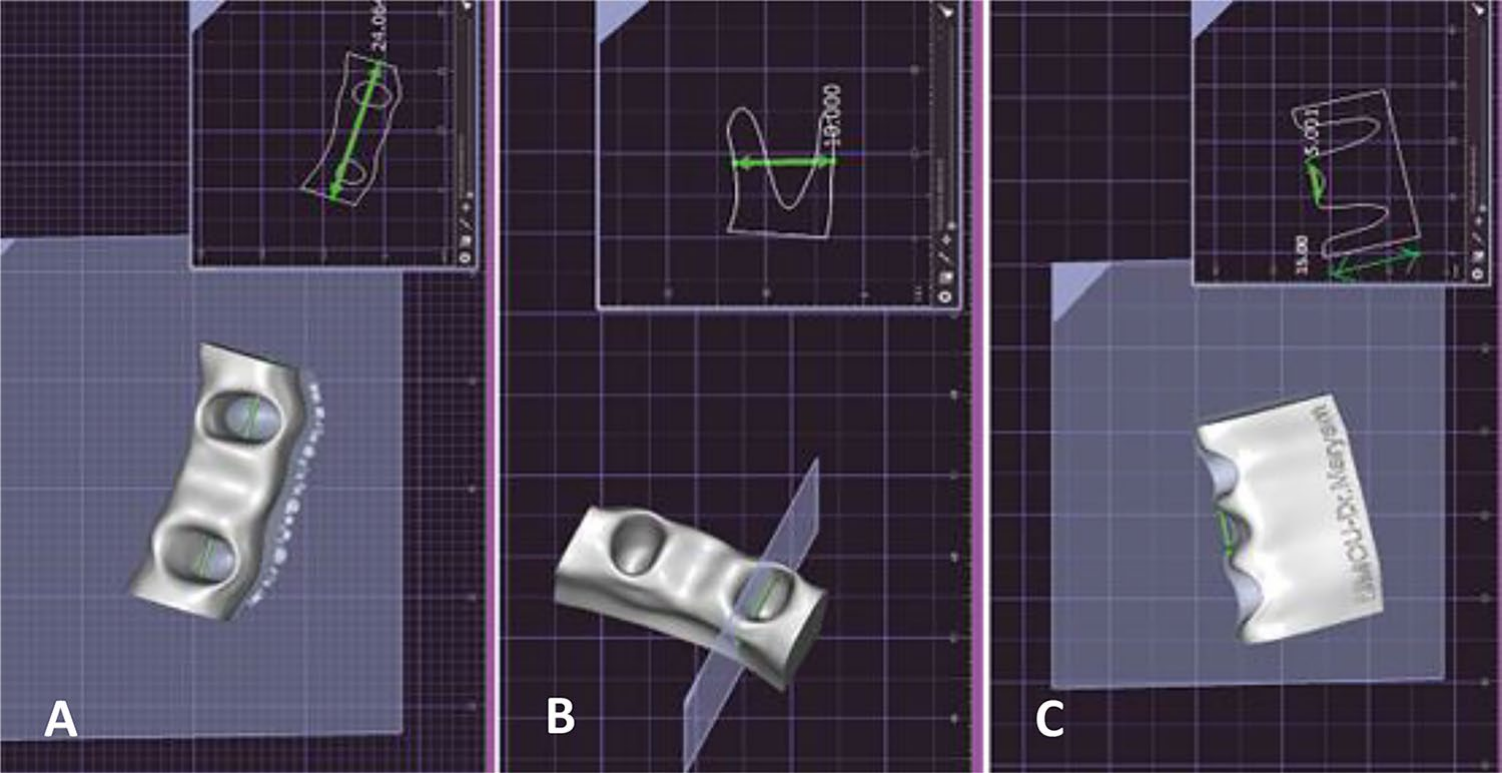
The base dimensions (24.00 mm × 10.00 mm × 15.00 mm). (**A**) Base length = 24.00 mm. (**B**) Base width = 10.00 mm. (**C**) Base height = 15.00 mm.

**Figure 2:**
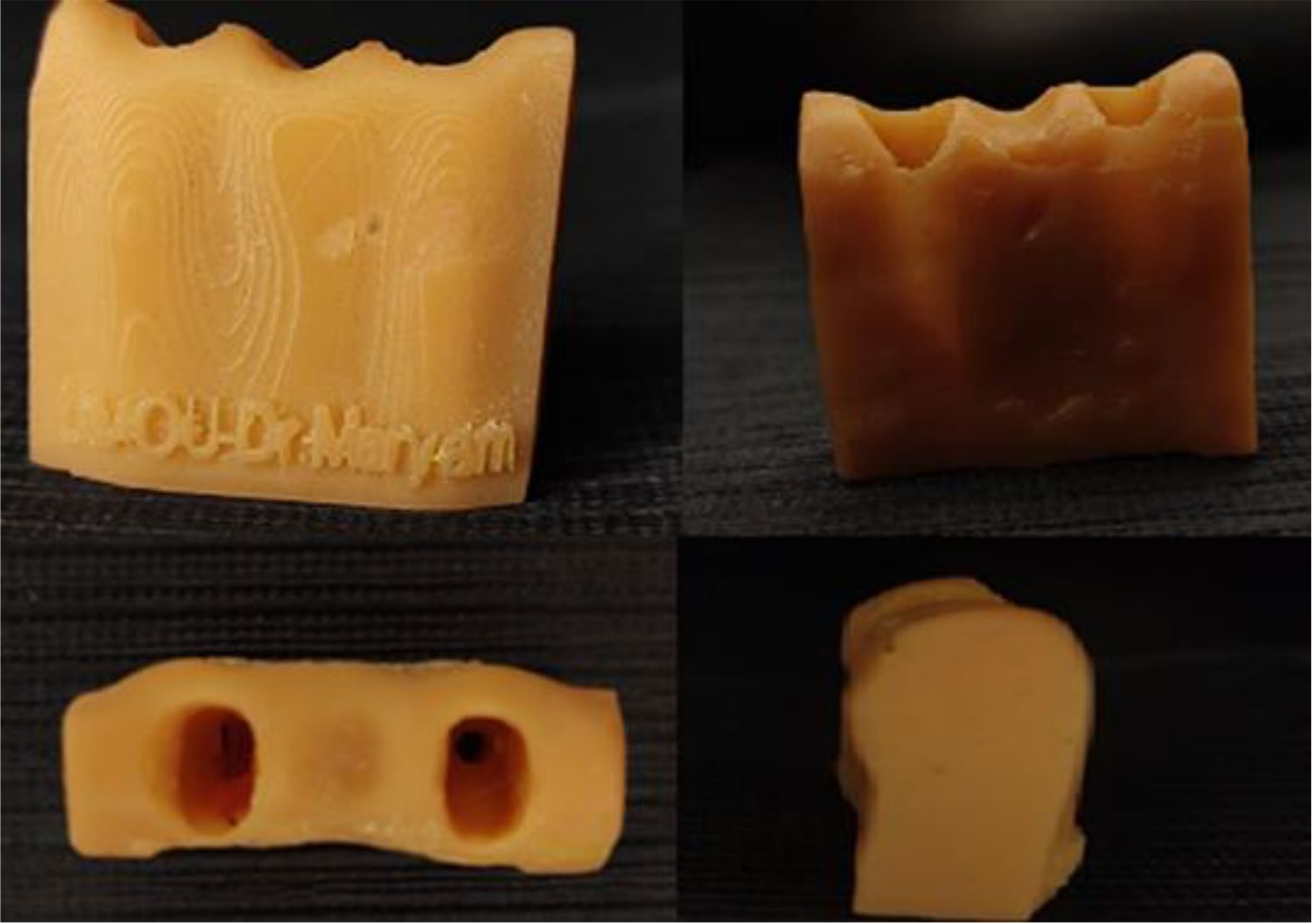
The printed base. Two holes were made on the upper face of the base, leaving a space between them similar to the width of a lower incisor, extending to the lower face. These holes should have a width similar to that of a lower incisor.

**Figure 3:**
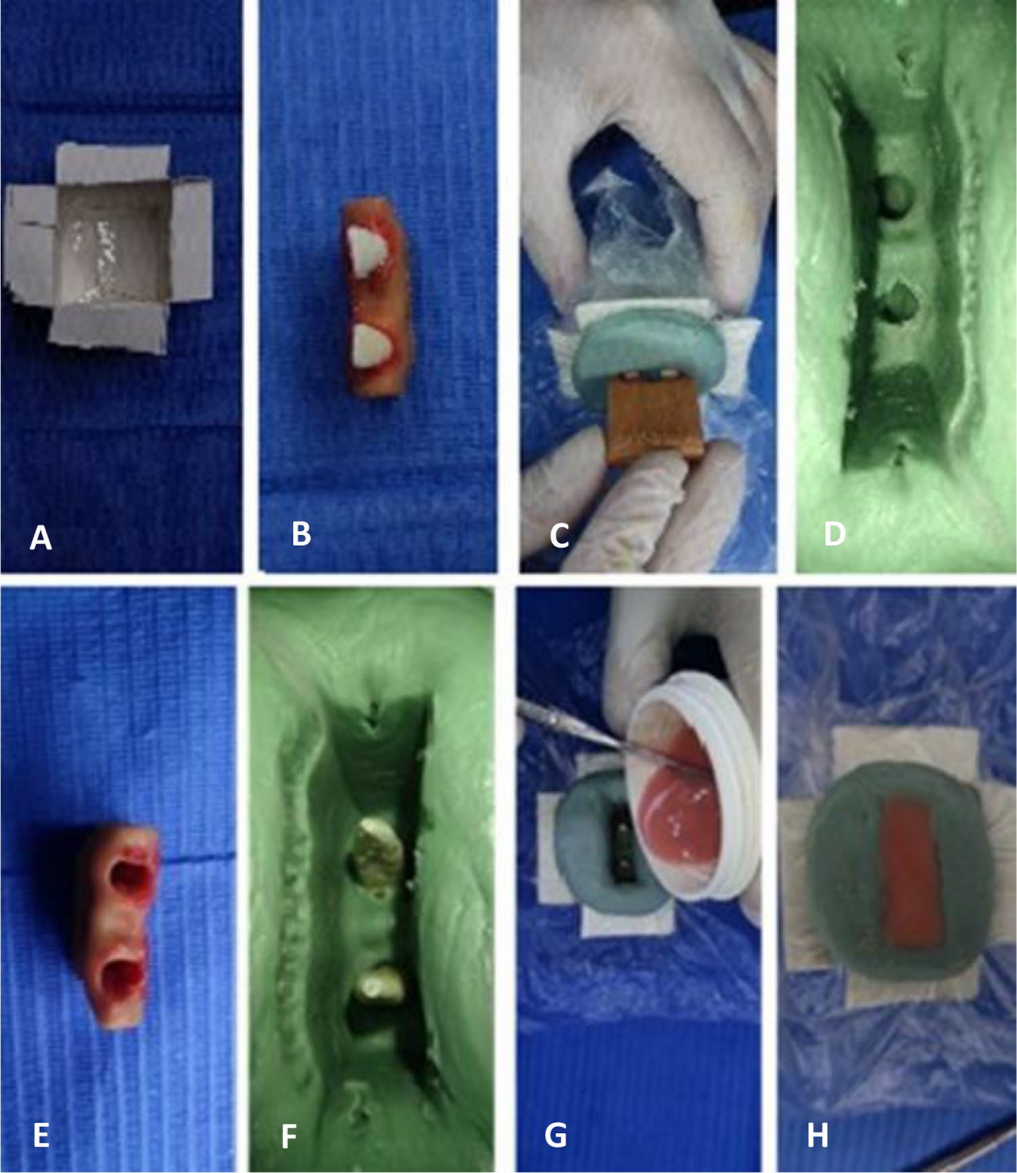
Teeth casting into an acrylic base. (**A**) The cardboard cube. (**B**) Fixing teeth inside the holes of the printed base using wax. (**C**) Condensation silicone was mixed to make an impression of the teeth and the resin base. The mixed condensation silicone was poured into the cardboard cube. (**D**) After the impression sets, the base and teeth were removed. (**E**) The printed base after the impression. (**F**) The teeth were cleaned then they are put back into their respective positions inside the impression. (**G**) Self-cure acrylic is mixed and poured into the impression, covering teeth roots. (**H**) The acrylic will set and form a solid base that holds the fixed teeth in place.

A virtual model resembling the anatomical structure of the mandible was digitally created using the exocad software (DentalCAD® 3.1 Rijeka, exocad, Hesse, Germany). The model was designed to have specific dimensions of 24.00 mm in length, 10.00 mm in width, and 15.00 mm in height. Two holes were incorporated on the top surface of the model with a gap between them equivalent to the width of a lower incisor, extending through to the bottom surface. These holes were sized to match the dimensions of a lower incisor (Fig. 1). Subsequently, the virtual model was transformed into a physical mold using a 3D laser printer (Shape X 3D Printer, Fusion technologies LTD., Beirut, Lebanon) and dental 3D printing resin material (Models, Fusion technologies LTD., Beirut, Lebanon) (Fig. 2). This printed base will serve as a mold to cast the teeth^10^. The teeth were securely positioned within the printed base using wax (Polywax, Bilkim Ltd. Co., Izmir, Turkey) to ensure that the teeth are securely held in place during the casting process. A condensation silicone mixture (zetaplus, Zhermack, Badia Polesine, Italy) was prepared to create an impression of the teeth and base. This mixture was poured into a cardboard cube, encasing the teeth and base. After the impression sets, the teeth and base were removed, cleaned, and repositioned the teeth in the impression. Self-cure acrylic (Millennium, Keystone Industries, New Jersey, United States) was then poured into the impression to create a solid base holding the fixed teeth in place. Once the acrylic had fully sets, the bases were carefully extracted from the impressions (Fig. 3). The acrylic bases were randomly divided into two groups, and bridges were mounted on each acrylic base. Each group consisted of 12 bridges as follows:

Group 1: computer-designed and manufactured mesh 3D bridges.

Group 2: sandblasted bridges.

#### Teeth preparation

**Figure 4:**
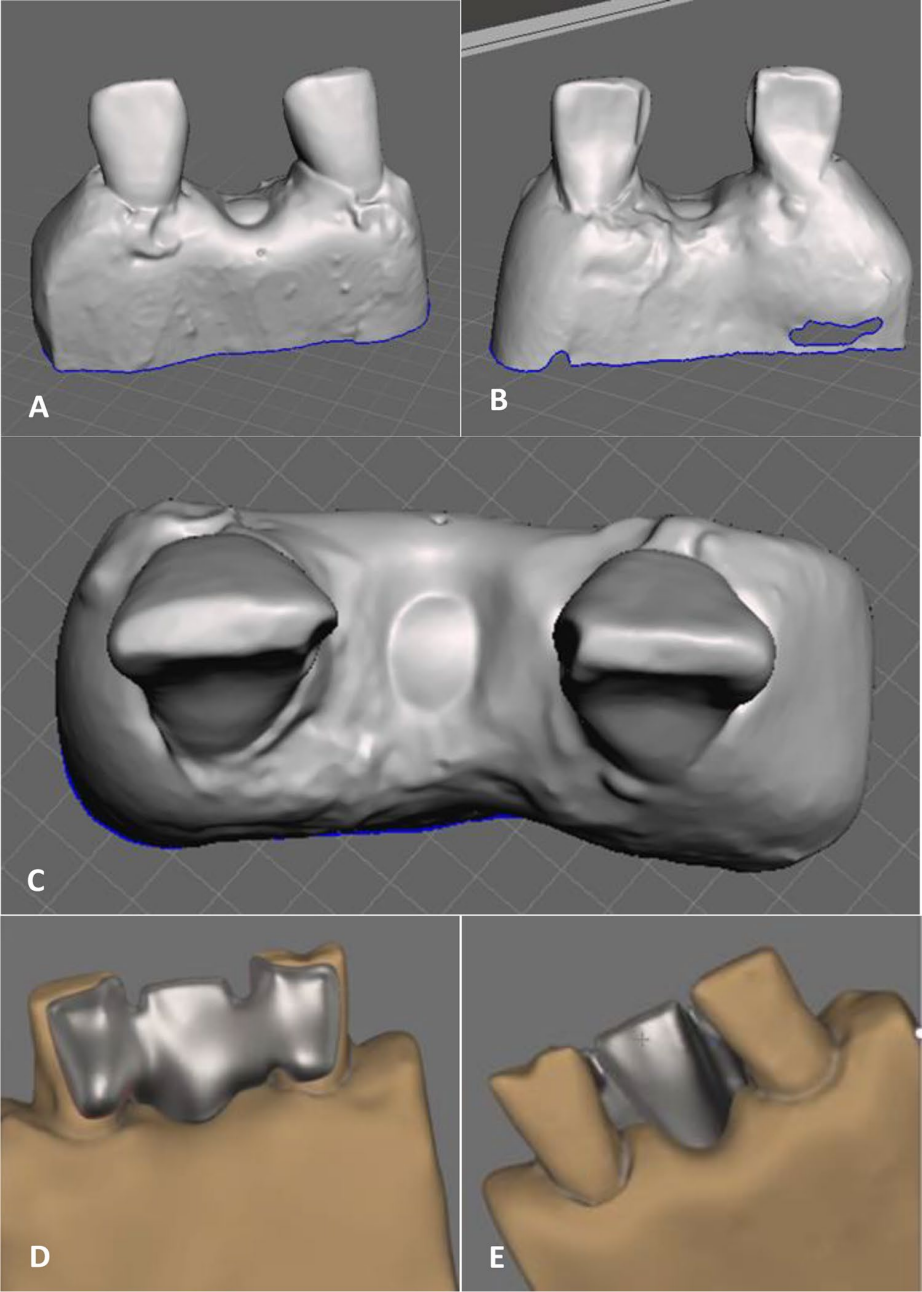
The teeth fixed to the acrylic bases were scanned using an extra-oral scanner to obtain a virtual dental cast. (**A**) The buccal aspect of the base and the fixed teeth. (**B**) The lingual aspect of prepared teeth. (**C**) The occlusal aspect of prepared teeth. (**D**) The bridge was created with wings, which extended over the lingual surface of the abutment and were positioned 0.50 mm away from the gingiva and the incisal edge. (**E**) The buccal aspect of the designed bridge.

The lingual surface was reduced by 0.50 mm. The preparation extended to 1.00–2.00 mm below the incisal edge and 0.50–1.00 mm above the gingival margin. The adjacent proximal surfaces to the edentulous area were prepared to provide a common insertion line for the two abutments using standard flat taper head diamond bur (Dentsply, Maillefer, Ballaigues, Switzerland) (Fig. 4)^8^.

#### Bridge design

**Figure 5:**
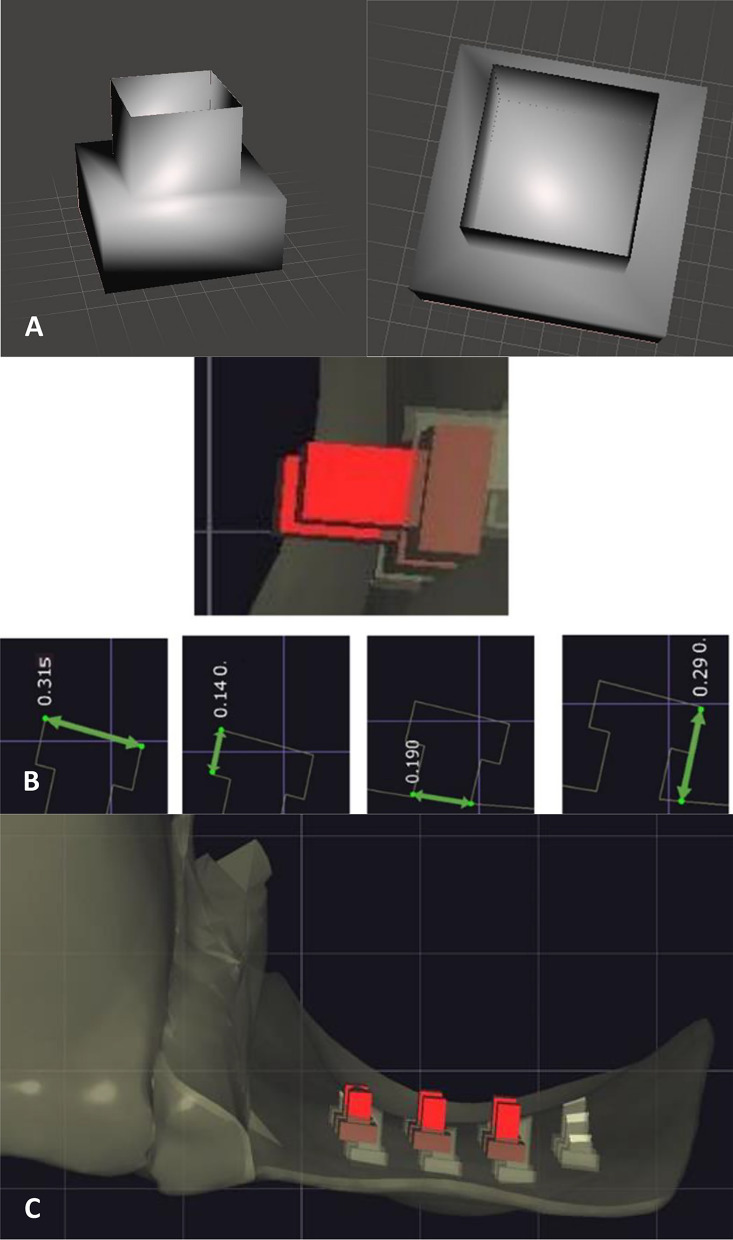
(**A**) A virtual tool is designed in the form of two rectangular prisms, one perpendicular to the other. (**B**) The virtual tool dimensions. The lower rectangular prism dimensions (315.00 μm × 315.00 μm × 140.00 μm). The upper rectangular prism dimensions (190.00 μm × 190.00 μm × 150.00 μm). (**C**) The holes alignment on the inside the wing using the virtual tool.

**Figure 6:**
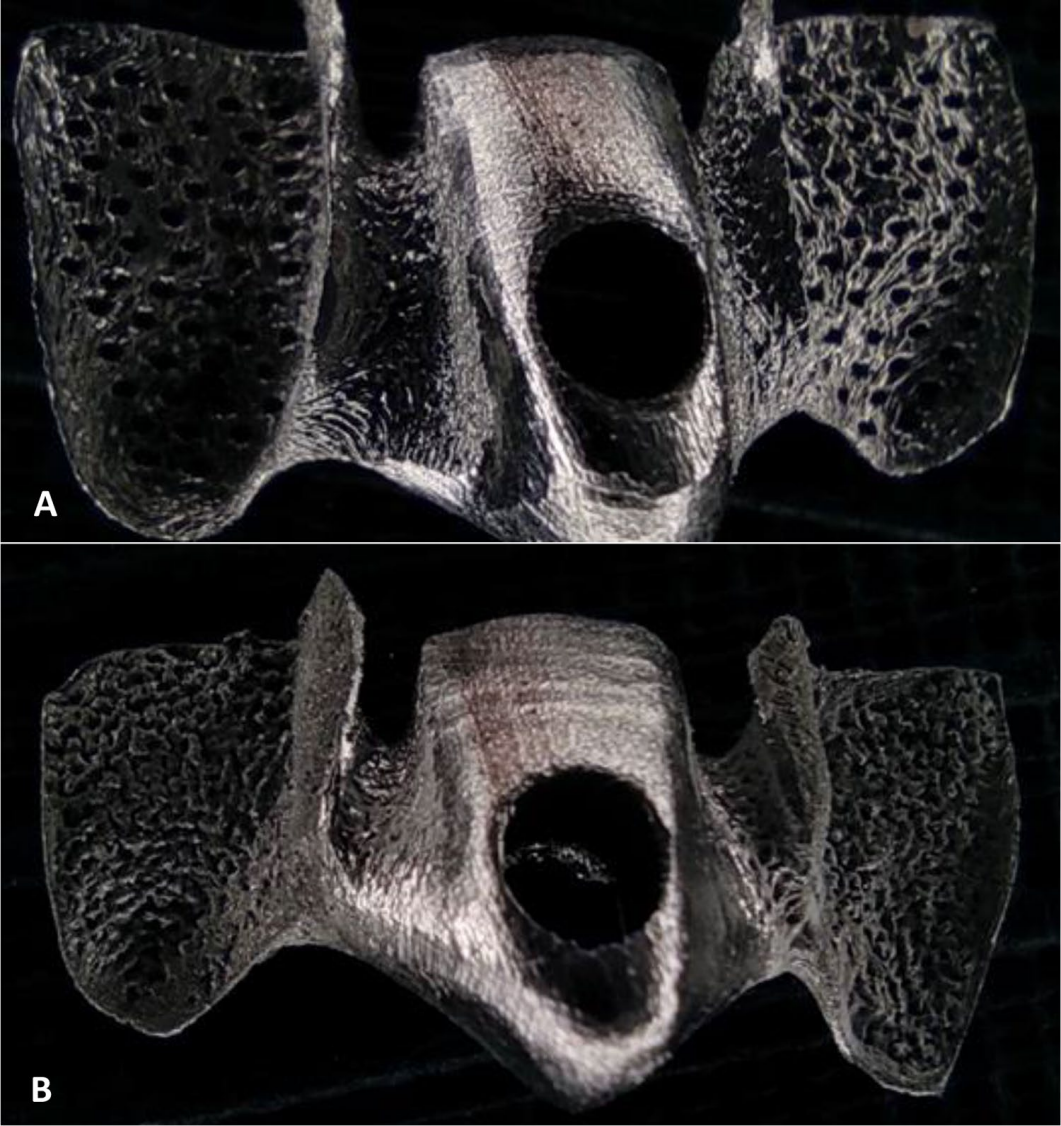
The bridges were printed using a 3D laser printer with an accuracy of 55.00 μm (**A**) 3D Mesh bridge. The holes were aligned to form a regular mesh shape inside the wing. (**B**) Sandblasted Bridge. Microscopic roughness and irregular holes are in the internal surface of the wing.

The abutment teeth were scanned utilizing a scanner (Medit T710, Medit, Seoul, Korea) (Fig. 4). Subsequently, virtual adhesive bridges were designed, incorporating wings that extended over the lingual surface of the abutment teeth positioned at a distance of 0.50 mm from both the gingival margin and the incisal edge. A central hole was created in the pontic to allow the insertion of a test wire. Micro perforations were strategically incorporated into the internal surfaces of the wings of 3D mesh bridges. These holes did not extend to the outer surface and were designed as two perpendicular rectangular prisms, with one prism nested within the other. The lower prism had dimensions of 315.00 μm × 315.00 μm × 140.00 μm, while the upper prism measured 190.00 μm × 190.00 μm × 150.00 μm. These intricate features were meticulously created using the virtual tool within the exocad program, which was developed using Autodesk software (Autodesk® Fusion 360TM, Autodesk, Inc., California, United States) (Fig. 5). The micro perforations within the wings of the 3D mesh bridges were strategically positioned with the smaller base oriented towards the lingual surface of the abutment tooth and the larger base facing the interior of the bridge. These holes were aligned, resulting in a regular mesh pattern within the wing structure (Fig. 5). These bridges were fabricated using a 3D laser printer (MYSINT100, SISMA USA Inc, New Jersey, United States) with a high precision of 55.00 μm (Fig. 6), utilizing cobalt chrome powder (realloy-classic, realloy e.K., Krefeld, Germany) as the material for 3D printing. Sandblasting was performed on the wings of the bridges using a stream of aluminum oxide particles with a size of 250.00 μm to create microscopic roughness and irregular holes on the internal surface (Fig. 6). The sandblasting process involved directing the particles at a distance of 1.00 cm for 15.00 s at a pressure of 6.00 bar. The external surfaces of the bridges were finished and polished for both groups^11^, while the 3D mesh bridges did not undergo the sandblasting treatment.

#### Teeth condition

The dental surfaces were pretreated by etching with 37.00% phosphoric acid (N-Etch, Ivoclar Vivadent, New York, USA) for 30.00 s. Subsequently, following rinsing with water and drying using oil-free air, an adhesive primer (multilink^®^ primer A + B, Ivoclar Vivadent, New York, USA) was applied to the dental surface for 1.00 min.

### Bridges condition and adhesion

The bridges were prepared by applying a cleaning paste (Ivoclean, Ivoclar Vivadent, New York, USA) to the metal surface for 60.00 s, followed by washing and drying the bridges and the application of a universal primer (Monobond plus, Ivoclar Vivadent, New York, USA) for two minutes. The adhesive bridges were affixed to their abutments using self-cure resin cement (Multilink Automix, Ivoclar Vivadent, New York, USA). The resin cement was applied to the inner surface of the bridge and the lingual surface of the abutment teeth. The bridges were placed in their proper position with finger pressure. Excess cement was removed using a dental probe, and a glycerine gel (Liquid Strip, Ivoclar Vivadent, New York, USA) was applied to prevent oxygen inhibition. Conditioning procedures adhered to manufacturer guidelines.

### Debonding test

**Figure 7:**
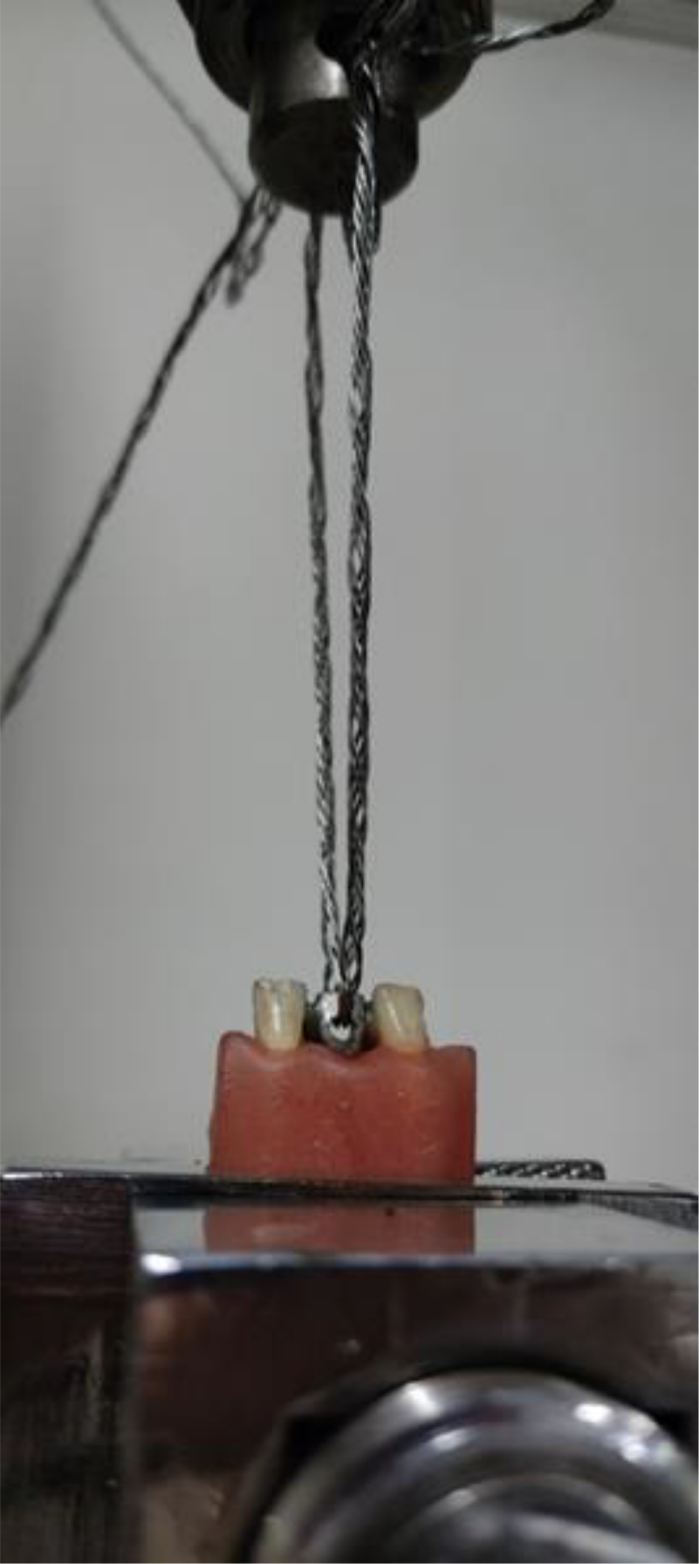
The debonding test was conducted against the insertion axis of the bridge in the upward direction. The resultant force were quantified in Newton (N) units.

After 24.00 h, a debonding test was carried out. The debonding test was executed against the insertion axis of the bridge utilizing a universal mechanical testing instrument (X100, The Testometric Co. Ltd., Manchester, United Kingdom) at a controlled speed of 1.00 mm/min in the upward direction until failure occurred (Fig. 7). The resultant forces from these assessments were quantified in Newton (N) units. The Kappa coefficient of intra-examiner reliability was > 0.8^9^.

### Statistical analysis

Statistical analysis was conducted utilizing IBM SPSS software version 24 (IBM SPSS Statistics^®^ version 24, IBM Corp., New York, USA). The independent samples t-test was employed at a 95% confidence level to analyze the outcomes. Descriptive statistics of bond strength were reported as mean, standard deviation (SD), standard error (SE), minimum (Min), and maximum (Max).

### Informed consent

Informed consent was obtained from all subjects.

## Results

Table 1 presents the descriptive statistics and outcomes of an independent samples t-test conducted to compare bond strength between two study groups. The mesh bridge group exhibited a mean bond strength (387.89 N; SD = 24.15), while the sandblasting bridge group had a mean bond strength (161.46 N; SD = 31.25). The statistical analysis revealed a significant difference in bond strength values between the two groups at a 95% confidence level (p < 0.05) (Table 1).

**Table 1:** Descriptive statistics and the results of the independent samples t-test for comparison of bond strength between study groups.

Groups	n	Bond strength					Independent samples t-test		
		Mean	SD	SE	Min	Max	Mean difference	t-Value	p-value
Mesh bridge	12	387.89 N	24.15	6.97	349.90	440.50	226.43	19.86	< 0.001
Sandblasted bridge	12	161.46 N	31.25	9.02	113.00	222.40			

The resultant force were quantified in Newton (N) units.

## Discussion

Many physicians tend to avoid using adhesive bridges due to their documented high rate of failure, as evidenced by multiple research studies^3,12^.

Despite this, adhesive bridges offer a swift, minimally invasive, and visually appealing treatment alternative when other options such as, implants or traditional bridges are not suitable, particularly for the replacement of a single tooth^13^. Consequently, there remains a demand for bridges that are conservative, easy to fabricate, more stable, and longer-lasting. Recent advancements in dental tools and methodologies have facilitated the creation of novel bridge designs with simpler production techniques and enhanced precision^14^. The computer-designed mesh bridge was specifically designed for this study to address the challenges encountered with older adhesive bridges, and its efficacy in terms of retention needed to be compared with a standard method. Hence, the objective of this study was to assess the retention capabilities of a mesh bridge, which was designed and manufactured utilizing computer technology and printed using a 3D laser printer, against the aluminum oxide sandblasting technique.

The lower anterior teeth are the smallest in size and surface area in the jaw. It is essential to maintain dental tissue integrity, and this necessitates exploring alternative treatment modalities like adhesive bridges when one of these teeth is lost. Freshly extracted human mandibular anterior teeth were chosen based on orthodontic indications or periodontal disease. Only sound teeth free of caries, fractures, or structural abnormalities and not subjected to prior treatments were utilized to guarantee adequate enamel surface for resin cement bonding^8^. To preserve the extracted teeth, they were immersed in a 0.50% chloramine T solution. This specific concentration was selected by established protocols for tooth preservation, as it effectively inhibits microbial growth without compromising adhesive properties^9^.

To enhance precision beyond traditional tooth casting methods utilized in certain studies^8,15^, a virtual design of a resin base was generated and subsequently fabricated using 3D printing technology. This resin base functioned as a template for embedding the teeth. The primary objective of this innovative technique was to achieve uniform inter-tooth spacing around the base, thus mimicking the clinical environment with greater fidelity. The axial groove preparation method was employed to standardize the insertion line and ensure precise placement of the bridge^16^. Various metal surface treatment techniques were utilized to enhance the mechanical retention of metal adhesive bridges, including perforation in Rochet Bridge, lost salt and acrylic granules methods, electrolytic etching in Maryland Bridge, and traditional Mesh bridge^17,18^. The exocad software was utilized to design virtual adhesive bridges with small retentive holes of precise dimensions and unique shapes to align with advancements in dentistry^10,14^. The holes were strategically arranged in a regular mesh pattern on the internal surface of the bridge wings, with the smaller base oriented towards the abutment tooth and the larger base facing the inside of the bridge. This configuration facilitated resin cement penetration into the holes, preventing its escape and enhancing resistance to debonding and lateral forces. Notably, this design offered precise control over dimensions, size, and shape. Furthermore, the computerized design was produced using a 3D laser printer, streamlining the process and introducing an innovative surface treatment approach for adhesive bridges to enhance their retention. The sandblasting technique was selected as the reference standard for comparative analysis due to its proven efficacy in augmenting bond strength. This method offers various advantages, including thorough surface decontamination and elimination of impurities. Additionally, it alters surface energy by generating microscopic roughness^19^, thereby improving surface-wetting characteristics. This microscopic roughness creates irregular tiny recesses on the metal surface, further enhancing the bonding between the cement and the metal^20^. Moreover, sandblasting is a cost-efficient and straightforward procedure that does not necessitate advanced expertise. It can be applied universally to various metal alloys commonly utilized in dental applications^21^. The visible alteration in surface texture post-sandblasting serves as a clear indicator of its effectiveness^20^. The utilization of 250.00 μm aluminum oxide particles in the sandblasting process was found to be optimal, resulting in larger and more profound cavities compared to 50.00 μm particles^22^. This particle size demonstrated superior outcomes in bolstering the bond strength between the adhesive cement and the metal interface^23^.

Chemical-cured resin cement has been employed similarly in numerous studies for bonding samples to their abutments. This self-curing cement does not necessitate light activation, making it advantageous for applications involving metal bridges that obstruct light transmission. The debonding test was utilized in this investigation to evaluate the retention of bridges, a commonly accepted method. The samples underwent debonding test resembling this conducted by Narwani et al.^8^. Evaluating the prostheses' resistance to forces attempting to displace them along the insertion axis is considered the most effective way to assess their retention^8^. The bond strength of the 3D mesh bridge group exhibited a notable increase of nearly 2.50 times when compared to the aluminum oxide sandblasting bridges group. This substantial disparity in bond strength highlights a significant difference between the two groups. The enhanced bond strength of the 3D mesh bridges can be attributed to their unique characteristics, including specific shape, precise size, and well-aligned mesh hole patterns designed using computer-aided technology. These features create a retention form that facilitates the infiltration of adhesive cement into the mesh holes, ensuring deep penetration and effectively resisting prosthesis dislodgement during debonding test. The sandblasting method yielded superior bond strength compared to the perforation technique as reported in previous literature^8^. In contrast, the 3D mesh bridge approach demonstrated significantly higher bond strength relative to the sandblasting method using aluminum oxide particles, with an approximately 2.50 times increase in bonding force values. The enhanced bond strength observed with the 3D mesh bridge approach compared to traditional sandblasting techniques may be attributed to the precise size and distribution of the mesh holes on the surface, which create a more effective retention form. The smaller nozzle size of the mesh holes, along with their internal positioning within the structure, allows for better retention of the resin cement within the holes, leading to increased resistance against debonding forces and improved stability. Furthermore, the design differences in preparation methods between previous studies and the current study, which use grooves that standardized insertion lines in the current study, could also contribute to variations in retention values^16^. The synergistic approach of combining a mesh structure with specifically designed holes resulted in superior bonding strength compared to using either the mesh or holes in isolation. Similar to the current study, the method employed in this study involved strategically placing holes on the bridge surface that did not extend to the outer surface, forming a pattern conducive to accommodating the mesh structure. These holes were characterized by precise dimensions and distribution, leading to significantly enhanced stability values. Albert et al.^15^ elucidated that incorporating two macro-retention techniques on the internal surface of the bridge wing contributed to the notable increase in bonding strength values.

This in vitro study's findings may not directly translate to real-world clinical scenarios within the oral cavity due to various factors such as lateral forces, repeated compressive forces, fluctuations in saliva pH, and temperature changes^8^. These environmental variables could potentially influence the outcomes observed in this controlled laboratory setting. Therefore, it is essential to exercise caution when applying these results to clinical practice. Future clinical studies are recommended to assess the extended-term efficacy of adhesive bridges employing diverse metal treatment approaches.

## Conclusion

Within the limits of this in vitro study, it is deduced that utilizing a computer-designed three-dimensional mesh technique produced by a 3D laser printer led to a substantial enhancement in bond strength values when compared to the traditional aluminum oxide sandblasting method. The retention achieved with the mesh method was approximately 2.50 times greater than that of the sandblasting approach. The adoption of this innovative technique has the potential to enhance the durability and efficacy of adhesive bridges over an extended period.

## Data Availability

The datasets generated during and/or analysed during the current study are available from the corresponding author on reasonable request.
